# Listening to Stakeholders’ Voices on Funding Social Inclusion in Sport for People with Disabilities—Proposal for Criteria

**DOI:** 10.3390/sports12060147

**Published:** 2024-05-27

**Authors:** Maria João Campos, Viktorija Pečnikar Oblak, Alain Massart, Predrag Ljubotina, Szilvia Perényi, Judit Farkas, Hugo Sarmento, Mojca Doupona

**Affiliations:** 1 Faculty of Sport Sciences and Physical Education, University of Coimbra, 3040-248 Coimbra, Portugal; alainmassart@fcdef.uc.pt (A.M.); hugo.sarmento@uc.pt (H.S.); 2 Research Unit for Sport and Physical Activity (CIDAF), 3040-248 Coimbra, Portugal; 3 Faculty of Sport, University of Ljubljana, 1000 Ljubljana, Slovenia; viktorija.pecnikar-oblak@fsp.uni-lj.si (V.P.O.); predrag.ljubotina@fuds.si (P.L.); mojca.doupona@fsp.uni-lj.si (M.D.); 4 School of Advanced Social Studies Nova Gorica, Gregorčičeva ulica 19, 5000 Nova Gorica, Slovenia; 5 Department of Sports Management, Hungarian University of Sports Science, 1123 Budapest, Hungary; perenyi.szilvia@tf.hu (S.P.); farkas.judit@tf.hu (J.F.)

**Keywords:** inclusive sport, sport finance, mainstreaming

## Abstract

The value of sport is extensively documented; however, there are still groups who do not have the opportunity to enjoy the benefits of sport due to lack of investment, particularly important for people with disabilities. A gap persists in understanding the effectiveness of inclusive sport programs in addressing equity-related targets, particularly on the effective methods of financing inclusion in sport for individuals with disabilities. Therefore, providing a platform for stakeholders to express their perspectives becomes crucial. Through focus groups and the World Café approach, the aim of this study was to gather insights from athletes, parents, professionals, and sport club managers regarding the funding of inclusive sport for people with disabilities. A total of 72 participants took part in nine focus groups in Portugal, Slovenia, and Hungary: 27 athletes with disabilities, 22 coaches, other technicians and parents, and 23 club managers/directors. Findings were divided into five topics: (1) perspectives on current funding satisfaction; (2) perspectives on sources and criteria for funding; (3) perspectives on ideal funding; (4) perspectives on ideas to reach decision-makers; and (5) proposals on ideal criteria for funding. Based on these findings, six measurable criteria for fair funding allocation were suggested that could develop a properly weighted system of criteria for decision-makers to assess the allocation of funding among inclusive sport organizations with the potential to catalyze broader policy and societal changes. Additionally, there is a pressing need to develop a funding model for inclusive sport for individuals with disabilities.

## 1. Introduction

Sport has the power to serve as a catalyst when it is shaped to improve people’s opportunities, confidence, and sense of belonging in the short run while laying the ground to change mindsets and build inclusive societies [1].

According to the Council Conclusions on access to sport for people with disabilities [2], not only are sport and physical activity a basis for personal, social, and learning development, but they also motivate social inclusion and integration. Sport and physical activity promote tolerance, solidarity, inclusiveness, and many other sporting and European Union (EU) values. Sport also provides people living with a disability an opportunity to increase their participation in society, showcase their talents, and challenge stereotypes. Sport’s values in relation to social inclusion, and in particular the role of sport in promoting and achieving the integration of minority and marginalized groups, are also recognized.

Social inclusion in sport is a modern concept that is gaining ground; despite many attempts and good practices, it is still in its beginning. Based on a review of existing articles, an attempt to define social inclusion in sport was recently developed [3]:

Social inclusion in sport for people with disabilities is a key approach that ensures all individuals, regardless of their abilities/disabilities or backgrounds, can actively participate in sporting activities within mainstream sport organizations. Coaches cater to individual needs, promoting mixed-ability activities to provide equal opportunities for athletes and other participants like volunteers. The benefits include enriched experiences, improved skills, and a sense of belonging. Researchers’ findings and coaching outcomes support this approach, revealing its positive impact on participants. However, obstacles such as exclusionary norms, attitudinal barriers, and inadequate policies may hinder full participation.

According to the authors, further progressive work is needed to define social inclusion in sport based on the existing literature, particularly in terms of support for social inclusion policies in sport, with one of the key pieces of information being the absence of measurable criteria for guiding policy funding in this domain. Thus, one of the areas of intervention needed is financing sport for policy coherence and grassroots impact: Within this topic, the United Nations (UN) report states that, on one hand, the private sector can and should increasingly help to deliver sport for development and peace, and, on the other hand, the paradox of sport is that those with access to the largest pool of players (grassroots sport) have the fewest resources. Community-level actors implementing sport for development and peace are often dependent on public and corporate funding [1]. Moreover, the targets of policy-level interventions have not been well studied among people with disabilities, and the effects of such policy changes on the physical activity levels and well-being of people with disabilities have not been examined [4,5].

The standards, conditions, and criteria for assessing and financing inclusion in sport needs to be well defined, as current systems focus on the majority of competitive sport, where the indicators of mass participation and competitiveness count above all, considering the number of medals earned. However, given the relatively smaller population of people with disabilities, the emphasis on mass participation does not hold the same weight. Inclusion efforts aim for broader objectives such as providing equal program options, fostering socialization, and promoting normalization, which require different evaluation criteria beyond sheer numbers or competitiveness in sport [6].

As for the criteria for funding inclusion in sport, the scientific literature is limited, and to the best of our knowledge, there is no existing article that specifically addresses this theme for people with disabilities. The European Commission report (European Commission, 2018) [7] reveals the complex range of sources and varying governance and management structures among the member states and has made it challenging to determine the overall funding levels for disability sport participation. In some nations, funding is channelled through NGOs or Paralympic committees and associations, while in others, key programmes are delivered directly by national sport agencies or ministries for sport, health, or education. Therefore, identifying funding criteria specifically tailored to include people with disabilities within the sport context is crucial. These criteria should address the unique needs and challenges faced by individuals with disabilities, ensuring that the allocated funds effectively support and promote their active participation in sport. The report also underscores the necessity of comprehending the most effective methods to finance inclusion in sport for individuals with disabilities. Thus, granting a platform for stakeholders to express their perspectives becomes pivotal in this pursuit.

Therefore, in disability research, according to Kroll et al. (2007) [8], focus groups have the potential to elicit individuals’ perspectives, experiences, and preferences across diverse settings and topics. These qualitative research methods facilitate the collection of detailed information from multiple participants simultaneously, often empowering them to express their beliefs, observations, and experiences, especially when peers with similar characteristics are involved. Although qualitative methods are an integral and important part of research with groups that would otherwise be excluded, four aspects may pose considerable challenges to the focus group methodology when used with people with learning disabilities: the ability to respond and engage; the need for facilitators in discussion; the possible ‘over-researching’ of certain groups; and the lack of any immediate impact of research on quality of life issues for participants [9]. New approaches are needed for bringing communities together to inform local movements that could be carried out alongside system-level initiatives, and one approach that engages diverse community members in solution-focused discussions is the World Café [10]. The World Café method can considerably enrich the toolbox of qualitative researchers [11] since it is well suited to collect the views and perceptions of a relatively large group of people over a relatively short period of time and is resource-efficient. It helps in the exploration and verification of themes over a large number of participants, and it also benefits the participants as it facilitates dialogue and mutual learning [12].

Through World Café focus groups and a concept mapping approach, the aim of this study is, through the opinions of athletes, parents, professionals, and sport club managers, to propose funding criteria for social inclusion in sport for people with disabilities. These findings should offer a unique and innovative insight to investors, decision-makers, and stakeholders who are responsible for the development of inclusion policies and for the allocation for funding inclusive sport for people with disabilities, as they can implement those criteria and act as catalysts for greater policy and societal changes.

## 2. Materials and Methods

A convenience sample was used. The data collection method used three different focus groups: (1) athletes with neurodevelopmental disorders; (2) coaches, social workers, psychologists, other technicians, and parents/guardians; and (3) sport club managers/directors. The data collection used the World Cafe method (Brown, 2005) [13]. A total of nine focus groups were held, three in each partner’s country (Hungary, Portugal, and Slovenia), across sessions of around 3 h. This study was approved by the University of Coimbra Ethics Committee, number CE/FCDE—UC/0025 2023. Informed consent was obtained in written form by the participants or the participants’ parents or guardians.

### 2.1. Participants

A total of 72 participants took part in focus groups in Portugal, Slovenia, and Hungary. From the lot, 27 were athletes with neurodevelopmental disorders; 22 were professionals such as coaches, social workers, psychologists, other technicians, and parents/guardians; and 23 were club managers/directors from mainstream and specialized sport and other organizations running inclusive sport programmes. Table 1 describes each countries’ focus groups.

Inclusion criteria were related to the inclusive sport setting: athletes with disabilities and professionals and managers that work in mainstreaming sport clubs.

Athletes were mostly young or adult women and men aged between 16 and 66 years with various disabilities such as ADHD, autism, head injury, intellectual disability, mental health issues, trisomy 21, and visual impairment. 

### 2.2. Procedures

Using the World Café approach [10,11,12], facilitators encouraged discussion about research topics, and discussions were framed by five questions developed by the research group (Table 2). Questions 1, 2, and 3 related to participants’ perceptions regarding current funding for inclusive sport, question 4 was the key subject to obtain possible criteria for funding social inclusion in sport, and question 5 was intended to hear participants’ opinions on how can they support decision-makers in the implementation of the criteria.

The focus groups followed identical methodology and questions in the respective local languages of the three countries. Subsequently, all partners translated the responses into English, ordered them based on the participants’ perceived importance, and submitted them to the designated analysis group of experts. The objective of the data analysis from the various focus groups in the three countries was to refine the most significant opinions voiced by participants regarding funding for inclusive sport involving individuals with neurodevelopmental disorders. The data underwent separate analysis by three researchers, employing a unified methodology. Discrepancies were then discussed to reconcile differences and establish shared findings among them. Finally, the results were presented to the entire research group, allowing any remaining divergences in interpretations to be addressed and solved by the researchers. A thematic analysis to organize specific topics into broad thematic areas was used.

All the obtained results were divided into meaningful themes according to the principle of concept mapping. A cluster approach to concept mapping is a mixed-methods participatory research approach that integrates qualitative conceptual data and rigorous multivariate statistical analysis (SPSS version 28), transforming abstract conceptual data into visual representations or “maps” [14]. The second analysis in this approach is cluster analysis, which partitions the map of groups of statements or ideas into clusters [15,16]. The participatory nature of group concept mapping, as well as its fully integrated mixed-methodology, makes it a particularly useful, valid, and reliable methodology for public health research [14,17,18].

## 3. Results and Discussion

The aim of the present study was to ascertain athletes, parents, professionals, and sport club managers’ perceptions on funding inclusive sport for people with disabilities.

According to participants’ perceptions and opinions on the funding for inclusive sport for people with disabilities, the findings and discussion are divided into five topics: (1) perspectives on current funding satisfaction; (2) perspectives on sources and criteria for funding; (3) perspectives on ideal funding; (4) perspectives on ideas to reach decision-makers; and (5) perspectives on ideal criteria for funding.

In the different stakeholders’ focus groups (athletes, coaches/parents/technicians, and sport club or association managers), the answers were not very distinctive between the three countries, suggesting shared opinions and similar realities. Between the stakeholders’ focus groups, their views showed barely slight differences.

### 3.1. Perspectives on Current Funding Satisfaction

In terms of the overall thoughts towards the existing public funding model for sport involving individuals with neurodevelopmental disorders, the athletes in the focus groups across the three countries expressed greater contentment within their sport clubs or groups. They seemed satisfied with their practice conditions and attributed their well-being primarily to their coaches. According to the literature, coaches significantly contribute to the inclusion of people with disabilities due to their positive attitude and their ability to adapt training to meet individual athlete needs [19]. Consequently, coaches and sport participants often develop intimate and personalized relationships [20,21]. Moreover, when coaches prioritize equitable treatment among athletes, ensuring no disparities, friendships typically blossom significantly within the team [20,22]. While around a fifth of surveyed athletes acknowledge the benefits of public funding, they also perceive a discrepancy in financial support compared to mainstream sport. They express the necessity for additional funding to broaden their access to a broader variety of sport, acquire more equipment and resources, expand their training network, participate in international events across various categories, and alleviate the burden of fundraising to focus more on their training. Previous research [23,24] has already highlighted the lack of critical mass for sport competitions, limited sport options, and inadequately adapted competitions as barriers to sport participation for people with disabilities. Moreover, the unsuitable environments to accommodate their needs have been identified as another challenge [25,26].

Coaches, parents, technicians, and sport managers unanimously express dissatisfaction with the current public funding model for sport involving individuals with neurodevelopmental disorders. Their criticisms are particularly pronounced. Similar to athletes, they also note insufficient funding for this population but additionally highlight excessive bureaucracy, lack of transparency, inequality and inconsistency in criteria, and a lack of willingness to improve funding.

Managers go as far as expressing disappointment, stress, and demotivation due to state funding that appears to heavily rely on connections and acquaintances, coupled with insufficient information on application procedures, inappropriate deadlines, delayed feedback on fund allocations, and the necessity to navigate a complex array of requirements, often leading to resorting to unethical means to secure funds. The only positive funding source seems to be from international organizations and funding programs, such as Erasmus. This inadequacy in funding, as confirmed by all three groups, aligns with the existing literature, where inadequate funding is identified as a major barrier to sport participation and inclusion for people with disabilities [24,26,27,28,29,30]. Those complaints and challenges are corroborated by numerous authors who explain these difficulties by policies lacking clear definitions, explanations, and terminologies, leading to confusion and difficulties in interpretation and implementation [27,29,31,32]. Possible interference by ‘political arithmetic’ and ‘partisan support’ is also mentioned [33]. There is a clear need for a more comprehensive definition of social inclusion in sport for individuals with disabilities, enabling stakeholders to adopt a consistent approach to social inclusion in sport and thereby support all athletes in realizing their full potential, regardless of their abilities [3] as inclusion in mainstream sport is necessary to achieve such an “inclusive society” [6,34]. The literature also highlights a lack of willingness to change, often attributed to ableism within sport clubs [35], negative attitudes towards individuals with disabilities and their parents in mainstream sport [24,26,30], coaches’ beliefs that inclusion might disrupt training for non-disabled peers [36], or their superficial attempts to include individuals with disabilities [29].

### 3.2. Perspectives on Current Sources and Criteria of Funding

The primary sources of financial support for sport practice, as reported by athletes across countries, are predominantly parents or family members, followed by personal funds and support from institutions or clubs. Coaches, parents, and technicians also recognize parents as an important source of funding, but they highlight state subsidies from municipalities, tenders, and projects as the most cited sources. Additionally, they mention parental support, along with contributions from associations or federations (such as lottery funds, Special Olympics, and sport associations). Sport club or association managers highlight that programs for individuals with neurodevelopmental disorders are primarily financed through the club’s revenue-generating activities like membership fees and camps. Similarly, they also mention state funding, lotteries, associations, and federations, adding EU projects like Erasmus and sponsorships by companies employing parents. However, they do not consider parents as the main source of financing. Athletes uniquely refer to themselves as a financial support for sport practice, emphasizing the need for better funding to alleviate the burden of fundraising and focus more on training. Except for parents and sport clubs, which are significant sources of funds in certain groups, state funding, lotteries, associations, and federations are consistently referenced by all groups as sources of funds.

Interestingly, some parents mentioned a lack of awareness of available financing options, echoing findings from previous studies that highlight a lack of awareness among parents of children with disabilities regarding sport activities and financial resources [35,37], especially among individuals with intellectual disabilities [38]. Darcy et al. (2022) [24] suggested the need for a centralized database to provide accessible information on relevant grants and financial resources for parents and individuals with disabilities.

Regarding the criteria used for allocating public funds for sport practice among athletes with disabilities, the most commonly cited were the number of participants, the athletes’ success or skill level, and the level of health conditions. Notably, coaches, parents, and technicians mentioned that the category of sport practiced significantly influences subsidy allocation, with less popular sport receiving fewer subsidies and Paralympic sport receiving more funding, especially in comparison to unified/inclusive sport. This flagrant inequality in sport funding, as expressed by stakeholders involved in sport with people with disabilities, underscores the need to consider this in future policies.

Unified Sport, which involves young people with and without disabilities playing in mixed teams, has been shown to foster a sense of belonging, create new relationships, promote acceptance by peers, and facilitate friendships beyond the sport arena and also aids in securing employment in mainstream businesses [20,24,39], forming a basis for mutual identity among individuals with and without disabilities based on their shared sport experiences. The emotional and social benefits of Unified Sport sustain the participation of non-disabled players [40] and exemplify a mixed-ability approach that should be embraced more broadly in mainstream sport clubs, where it is currently in its early stages of implementation [3,6]. The disparity in funding for this successful and promising approach prompts a profound reflection on why the least funded strategy appears to be the most successful and promising, as supported by the opinions of coaches, parents, and technicians involved in sport for individuals with intellectual disabilities and neurodevelopmental disorders. Overall, there is a compelling need for fairer distribution of funding across different sport categories, recognition of the value of Unified Sport, and the implementation of a more inclusive approach in sport clubs to ensure equal opportunities and support for all athletes, regardless of their ability.

### 3.3. Perspectives on Ideal Funding

The inclusion of individuals with neurodevelopmental disorders in mainstream sport clubs often centres on adequate government funding, as concluded by several researchers, e.g., [24,28,29]. However, it is often observed that when clubs receive such funding, their primary focus tends to be financial gain rather than genuine inclusion. Consequently, many clubs aim to adapt minimally to fit the criteria for subsidies, often obtaining the same subsidies as genuinely inclusive sport clubs [36,41]. This underscores the need for more discerning criteria in funding sport.

The three groups involved in this study proposed several measurable criteria for a fair distribution of public funds for sport involving individuals with neurodevelopmental disorders. They emphasized factors such as the number of athletes, particularly focusing on young participants and their regularity of involvement (e.g., training hours and opportunities), the ratio of participants to coaches, the level of disability and specific technological or installation requirements, the frequency and quality of public events and media engagement, and the program’s connection with the community. Interestingly, all groups highlighted the importance of focusing on young athletes, aligning with the literature suggesting that younger individuals with disabilities who engage in sport with their nondisabled peers at an earlier age are more likely to reduce participation gaps [24,42].

Additionally, coaches and other technicians, parents, and athletes also proposed criteria such as the level of performance/success, adaptation to participants’ capacities, and participants’ financial capacity. Except for the athletes, the other stakeholders added that fair criteria should also include opportunities for lifelong exercise, support for long-term projects, operational years, budget utilization, the number and satisfaction of volunteers or involved individuals, and the impact of sport participation on the subjects. The literature emphasizes that inclusive sport programs should prioritize continuous training for physical, intellectual, emotional, and social development, not just participation [38]. On the other hand, only athletes highlighted criteria such as the number of organized competitions and the variety of sport offered. They emphasized the importance of joy, participants’ goodwill, access, and socialization for all athletes regardless of ability, suggesting these as significant criteria for funding distribution. However, performance results and competition organization were the main criteria cited for athletes. Parents, coaches, and technicians indicated that while competition should be considered, it should not be the primary criterion, and managers revealed that around 10% of members are able to compete and stressed the need for increased funding for non-competitive sport, especially among teenagers. This aligns with the idea that competition-focused activities may shift the emphasis from fun and participation to winning and competitiveness according to parental and coach opinions [24]. Activities that are more individualized, recreational, and less focused on success and competition tend to facilitate participation more easily [35,40].

As mentioned earlier, the primary reasons for athletes’ happiness in sport participation were coaches, positive feelings, meeting people, making friends, receiving support, and trying various sport, without explicit mention of competitive aspects. Therefore, a balanced approach that considers both competitiveness and quality of life and well-being aspects should be crucial in funding criteria. Fun, socialization, and skill-building competitions should be encouraged, offering ‘social’ competitions where everyone can participate. Creating more events where individuals with and without disabilities can participate together can encourage more clubs to engage in inclusive practices [24,36].

The focus group insights from coaches, parents, and technicians suggest several key criteria for fair funding allocation in sport for individuals with neurodevelopmental disorders. They propose considering the qualification level of coaches, the potential for employment opportunities within sport projects, and the regional interest in developing specific sport as important factors. Fair employment opportunities are crucial, given that individuals with disabilities face higher unemployment rates [37], often finding themselves in low-paying, low-skilled, and part-time positions [24,43].

The need for qualified coaches is extensively emphasized in the literature. For instance, for individuals with intellectual disabilities, the lack of professionals with appropriate training serves as a barrier to their participation in physical activities [6,38]; moreover, parents recognize a lack of awareness or knowledge among coaches regarding the inclusion of individuals with disabilities in sport and their adaptation of current sporting models to accommodate their needs [35,36,41]. Coach education and positive past inclusion experiences predict future involvement [32], showing that training opportunities can develop positive attitudes, disability-related knowledge, and interaction skills [20,42].

In order to adjust funding allocation, sport club or association managers propose using a coefficient that considers both sport results and non-competitive aspects. They suggest evaluating participants’ individual progress, encompassing various aspects like self-affirmation, self-image, self-confidence, skill development, personal growth, mental and physical health improvement, social acceptance, cooperation, empathy, and community strengthening, increasing expansion of the social network and assessing the satisfaction of the closest social network. This assessment could involve a customized questionnaire using descriptive criteria aligning with the literature indicating that physical exercise for people with disabilities leads to physical, skill-related, emotional, and health benefits, contributing to increased well-being, self-esteem, self-confidence, socialization, stress reduction, and personal growth. These are aligned with the literature, as sport and exercise may have a significant impact on how people with disabilities view themselves, helping them pursue greater challenges and strive for higher goals [22,44,45,46]. This personal growth and development empowers them to accept disability, overcome fears, and live life to the fullest [47], helping them develop the skills to become effective self-advocates and agents of change [32], giving meaning to their lives [30,37,40,47]. 

Moreover, engagement in organized sport enhances social contacts, interactions, networks, and friendships for individuals with disabilities, improving their quality of life and breaking down cultural, social, and linguistic barriers [22,41,48]. Social inclusion is underscored as crucial, emphasizing the need for society to recognize diverse ways of being human without prejudice, promoting complementarity and cultural diversity to empower individuals [25]. Studies suggest that mixed groups in sport benefit both individuals with and without disabilities (fitness, skill, enjoyment, psychosocial factors, sport meaningfulness), indicating shared preferences in activities [20,24,35,42]. Overall, sport projects and programs need support from government policies to enable social interaction between individuals with and without disabilities [6,49], since it can foster a more inclusive environment and promote the shared enjoyment and benefits of sport for all participants.

Comparison across the three distinct focus groups reveals both overlapping and distinct areas. Sociability and evidence-based documentation emerge as common themes. Athletes emphasized competitions and the variety of sport offered in the community, while coaches, parents, and experts underlined trainer development, consistent practice, work attitude, and the transformative impact of sport on participants. In contrast, managers and directors prioritized measurable criteria through documentation and process assessments via satisfaction questionnaires. They underscored inclusive settings within mainstream sport by establishing coefficients between trainee and coaching staff numbers and sport outcomes. Although acknowledging media support and ongoing operation, they emphasized experience, stability, and business sustainability.

### 3.4. Perspectives on Ideas to Reach Decision-Makers

The consensus among the three groups highlights the need to educate decision-makers through presentations, documentation, specific guidelines, and specific training sessions to implement essential criteria for funding sport for individuals with neurodevelopmental disorders. Additionally, influencing decision-makers via lobbying efforts from sport associations and other relevant entities is seen as crucial. Developing strategies for this endeavour involves creating connections between inclusive sport associations and other organizations while increasing visibility in society through media exposure and engagement with influential figures to help in changing minds. Studies indicate that increased societal exposure to individuals with disabilities leads to more favourable attitudes towards inclusion [20,42,50], emphasizing the importance of community education and information about disabilities to combat social exclusion [51].

Media coverage that portrays athletes with disabilities as athletes first, without sensationalizing or perpetuating stereotypes, is vital in changing public perceptions and advocating for policy measures that eliminate barriers and misconceptions [25,37]. However, there remains a gap in effectively marketing Paralympic sport in many countries, highlighting a need for improved strategies in this domain since the commercial interest in sport is guided by the proximity to spectators who become consumers and the desire of companies to approach these people to sell their products, with the role of marketing in the Paralympic sport market being of the first importance in this perspective, which fails in most of the countries [52,53,54].

Establishing effective networks is also crucial, as highlighted in various publications advocating for sport development projects, e.g., [35,55,56,57], claiming the implementation of transdisciplinary and intersectoral approaches involving social workers, allied health professionals, sport workers, and representatives of individuals with disabilities. This collaborative effort aims to address financial, organizational, and cultural challenges while reinforcing local-level activities for individuals with disabilities.

The athletes express a strong desire for managers and decision-makers to listen to their needs, advocating for better communication channels. Similarly, Darcy et al. (2022) [24] emphasize the importance of training in enhancing communication skills between individuals with disabilities and sport personnel such as coaches, referees, and officials, a crucial aspect often lacking and contributing to decreased participation rates. Moss et al. (2017) [51] demonstrate that inclusive changes, such as facilities designed with individuals with disabilities in mind, significantly increase participation rates to 29% of the membership. Athletes in the current study suggest greater involvement in the funding process, proposing membership in commissions for tenders, control over fund utilization, decision-making for assisting other athletes, and implementing criteria for funding allocation. The existing literature highlights that individuals with disabilities do not want to be labelled as incapable or reliant solely on societal solutions [24,58]; instead, they seek collaborative partnerships and engagement with policymakers and practitioners. Collaborations with the disability community have been proven productive in formulating strategies and proposing effective practices to sensitize stakeholders and policymakers [37]. Policy development should embrace participatory approaches, including input from individuals with impairments to engage their knowledge and experiences, ensuring their perspectives are incorporated into policy formulation and implementation [32,59]. Collaborations between disability sport organizations and non-disabled sport clubs, facilitated by policymakers, can give a voice to individuals with disabilities, leading to better monitoring, evaluation, and compliance by federations and clubs [37,59,60]. According to Lindsey et al. (2023) [61], there are striking gaps in policy research focusing on diversity and difference across young people and on increasing inclusion and integration in youth sport through policies that otherwise appear focused on increasing activity generally. These issues connect with the identified democratic deficit of not involving young people in policy-making.

Athletes focus groups also propose leveraging successful athletes to establish charity funds or charitable competitions without taxation. This approach is an interesting idea and aligns with research indicating that successful athletes with disabilities serve as role models, increasing social awareness and fostering engagement and participation [37], and that enhanced awareness can facilitate the establishment of accessible sporting facilities and organized sport programs for individuals with disabilities [51].

Parents, coaches, and technicians emphasize the need for uniform criteria and the establishment of a specific entity to oversee this task and they propose increased collaboration in club management and advocate for the support of a project manager to aid in the submission and implementation of tenders or projects. Furthermore, and alongside the sport managers, they stress the importance of motivating mainstream sport federations to embrace disabled sport, fostering greater inclusion in mainstream clubs and advocating for Unified Sport integration within Olympic committees. Specialists in the field emphasize the significance of mainstream sport federations collaborating with disability organizations, as this collaboration aims to build positive relationships, instil a sense of belonging beyond sport, foster mutual identity [62], and pool resources to develop innovative programs [36].

Many individuals with disabilities lack the confidence to participate alongside nondisabled peers, especially in the initial period after the onset of disability, engaging in a disability-specific sport setting to regain self-confidence, which later enables them to engage in non-disabled sport opportunities, creating a sport development continuum, as supported by Kiuppis (2018) [48], Kirakosyan (2019) [32], Klen et al. (2019) [63], Scifo et al. (2019) [45], and Christiaens and Brittain (2021) [36]. The access and utilization of public sport facilities and participation in community sport serve as essential performance indicators for measuring social inclusion through sport, as indicated by Chen and Liu (2020) [64]. While recognizing exceptions, the scientific community generally favours the mainstream sport club inclusion approach, e.g., [3], as it aims to provide individuals with disabilities equal access and success to sport and associated skill development, aligning with perspectives from Coalter (2015) [65], Geidne and Jerlinder (2016) [35], and Kirakosyan (2019) [32].

In summary, stakeholders have relevant ideas on how to reach decision-makers. Creating awareness, educating decision-makers, enhancing visibility through media and influential figures, improving marketing strategies, active involvement in decision-making processes and in policy development, and fostering collaborative networks are essential steps in advancing inclusive sport for individuals with neurodevelopmental disorders. These efforts collectively aim to eliminate barriers, promote inclusion, and reinforce support at both the community and policy levels, enhancing participation and creating more inclusive opportunities in sport for individuals with disabilities.

### 3.5. Proposal for Future Criteria in Funding Processes

After compiling, participants’ opinions on possible measurable criteria for funding data were divided into meaningful themes according to the principle of concept mapping [10]. From 410 responses in the focus groups, we obtained the 180 most important opinions, which were grouped in statements, using multivariate statistical analysis. Based on the data analysis and the keywords of the statements and on the examination of existing criteria in other contexts grounded in the literature, six final clusters (criteria) were defined, as outlined in Table 3.

The challenge of adequately funding social inclusion programs in sport is presented not only by the lack of a unified definition of social inclusion in sport [3] but also, according to some experts and decision-makers [72], by the mindset surrounding parasports. These receive a significant portion of funds from foundations primarily intended for the disabled population, not athletes. Thus, the so-called “disability sport” is somewhat of a hybrid, primarily financed by funds intended for the disabled, but also by applying for national and local funds meant for elite sport. Furthermore, the funds that foundations for the disabled allocate to sport are disguised under the guise of social programs, recreational sport activities intended for leisure activities and not elite sport [72]. The decision-makers believe that the funds are available but need to be properly reallocated and controlled in terms of spending and traceability. This can be achieved with the proposed measurable criteria for funding social inclusion programs in sport from Table 2. The study offers six options proposed by stakeholders, representing an innovation and progress in this field, especially considering that funds in sport are primarily distributed based on sport results (medals) and the number of participants in programs. When speaking of vulnerable individuals, we cannot guarantee either elite sport successes or mass participation. However, inclusion and a possibility to choose can be supported. The six proposed measurable criteria, (1) sustainability, (2) performance, (3) sociability, (4) attitude towards inclusion, (5) competencies, and (6) good governance, are intended for mainstream sport clubs, which can make significant progress through consistent implementation and adherence to these criteria and an inclusive approach, resulting in every village, town, and city having a dignified place for vulnerable individuals in sport. The sport club will be financially rewarded systematically for this but will have to prove it when applying for public funding. And precisely these six proposed criteria, on one hand, ensure that mainstream sport clubs can apply for funding and, on the other hand, provide decision-makers with control, spending, and traceability.

This study is not without limitations, and although qualitative methods are an integral and important part of research with groups that would otherwise be excluded, Kaehne and O’Connell (2010) [9] identified challenges to the focus group methodology when used with people with learning disabilities: the ability to respond and engage; the need for facilitators in discussion; the possible ‘over-researching’ of certain groups; and the lack of any immediate impact of research on quality of life issues for participants. Despite the many difficulties that researchers need to address when conducting focus groups with people with disabilities, the authors hold the view that a careful balancing of methodological rigor and a keen awareness of the limitations of focus group research can result in gathering valid data on a wide range of issues relevant to people with disabilities. While the World Café was not created as a research tool, a growing number of researchers have used the model to examine issues affecting the lives of people with disabilities [73]. Moreover, using a convenience sampling can affect the reliability and generalizability of the findings.

The views of each group highlight the multifaceted and complex nature of inclusive sport funding, which requires a comprehensive approach that brings together different viewpoints for holistic support and sustainable development in this key area. Moreover, there is a need to create a model for funding inclusive sport for people with disabilities. Although the insights of the participants suggest potential measurable criteria for funding social inclusion in sport, the need for further analysis remains. This would need to be supported by the vision of experts in a wider debate with national experts and decision-makers, which is necessary to reach a meaningful and widely accepted consensus on this hot topic. Besides the development of a funding model, future studies should include qualitative research with experts and decision-makers.

## 4. Conclusions

Through this study, we gather the insights of several stakeholders by hearing their voices about the current and the ideal conditions of inclusive sport settings, mainly on the criteria that support the recreational or sport for all initiatives. We have made significant theoretical contributions regarding the description of criteria and indicators to funding allocation: sustainability; performance; sociability; attitude towards inclusion; competencies; and good governance. These findings offer a unique and innovative insight to investors, decision-makers, and stakeholders who are responsible for the development of inclusion policies and for the allocation for funding inclusive sport for people with disabilities, as they can act as catalysts for greater policy and societal changes.

Our findings suggest a need for continued research to understand how those criteria could be effectively implemented. For this set of criteria to be enhanced and validated, it is imperative to conduct assessments across multiple countries, through the development of a questionnaire designed to assess the sport club settings. All relevant stakeholders, including sport managers, club members, and staff, must actively participate in this assessment. The involvement of all these parties is crucial to provide decision-makers with tangible and measurable criteria for the effective allocation of funding.

Based on the findings, six criteria are suggested for a fair funding allocation, since they can act as catalysts for greater policy and societal changes. Practical implications include the development of a sustainable model on a national level that can implement a properly weighted system of criteria for decision-makers in determining the share of funding among inclusive sport organizations. This model should enable comprehensive and sustainable support for different sport and organizations, contributing to more effective and equitable funding for inclusive sport activities.

## Figures and Tables

**Table 1:** Focus group characteristics.

Country	Focus Group	Facilitators	Participants	Female	Male
Hungary	Professionals	1	10	6	4
Hungary	Athletes	2	6	1	5
Hungary	Managers	2	8	1	7
Portugal	Professionals	1	3	2	1
Portugal	Athletes	3	11	4	7
Portugal	Managers	1	7	3	4
Slovenia	Professionals	1	9	6	3
Slovenia	Athletes	3	10	2	8
Slovenia	Managers	2	8	5	3

**Table 2:** Focus groups questions.

	Question
1	How are your sport programs for people with intellectual disabilities and developmental disorders financed?
2	What do you know about the distribution of public funding within the scope of sport for people with intellectual disabilities and developmental disorders?
3	Are you satisfied with current public funding models for sport for people with intellectual disabilities and developmental disorders?
4	What criteria should, in your opinion, be used for a fair distribution of public funds for sport for people with intellectual disabilities and developmental disorders?
5	How can you help decision-makers to implement those criteria?

**Table 3:** Recommended measurable criteria for funding inclusion in sport for people with disabilities.

Criteria	Description of Criteria Indicators	Supporting Literature
Sustainability	Sustainability as a value of sport can be identified through the number of members involved in a sport organization, number of years that it operates, the mission and goal quality, and access to facilities and to specific documentation.	[66,67]
Performance	Performance of sport organizations can be described, for example, by the number of inclusive sport events and competitions organized and participated in.	[68]
Sociability	Sociability may be raised attention to the social embeddedness of sport organization in the implementation of inclusive activities and social programs, in creating partner’s network.	[68,69]
Attitude towards inclusion	Attitude toward involvement of specific personnel ensuring the realization of inclusion in aspects of equality, ethics, safeguarding, and volunteerism, including regulations and written documents in these matters.	[68,69]
Competencies	Competencies are viewed through the sources of a sport organization in terms of qualified sport professionals to work with diverse abilities, quantity of inclusive training sessions, involvement in sport associations, and services provided to members or parents.	[70]
Good governance	Good governance may refer to the dimensions of inclusivity, fairness, transparency, legitimacy, accountability, direction, and capability.	[71]

## Data Availability

The data presented in this study are available on request from the corresponding author due to privacy and ethical reasons.
